# Hypertension at 5 months postpartum in women with gestational diabetes

**DOI:** 10.1002/uog.70291

**Published:** 2026-07-17

**Authors:** C. Gómez Fernández, M. Charakida, M. Moser, R. Breiviene, T. Mansukhani, L. A. Magee, P. von Dadelszen, K. H. Nicolaides

**Affiliations:** ^1^ Harris Birthright Research Centre for Fetal Medicine King's College Hospital London UK; ^2^ Faculty of Medicine Complutense University of Madrid Madrid Spain; ^3^ School of Biomedical Engineering and Imaging Sciences, King's College London London UK; ^4^ 2nd Department of University Paediatric Clinic National and Kapodistrian University of Athens Athens Greece; ^5^ Department of Women and Children's Health School of Life Course and Population Sciences, King's College London London UK

**Keywords:** adiposity, diastolic dysfunction, dysglycemia, dyslipidemia, echocardiography, hypertension

## Abstract

**Objective:**

Gestational diabetes mellitus (GDM) is associated with future maternal cardiovascular disease, independent of the development of Type‐2 diabetes mellitus. As a major cardiovascular risk factor, we examined the incidence and predictors of hypertension at 5 months postpartum in women with previous GDM.

**Methods:**

Between September 2023 and January 2025, we conducted a single‐center observational prospective cohort study of all women who received routine prenatal care at 12 weeks' gestation at King's College Hospital, London, UK, and developed GDM during the index pregnancy. Those with chronic hypertension were excluded from the analysis. Women were invited to a 5‐month postpartum visit, at which assessment was undertaken for hypertension (systolic blood pressure (BP) ≥ 130 mmHg or diastolic BP ≥ 80 mmHg, or receiving antihypertensive treatment), obesity, adiposity, dysglycemia, dyslipidemia and renal dysfunction. Logistic regression was performed to assess predictors of postnatal hypertension.

**Results:**

Among the 678 included women with previous GDM in the index pregnancy, 179 (26.4%) developed hypertension at a median of 5.1 (interquartile range (IQR), 4.4–6.7) months postpartum. Multivariable analysis showed that postpartum hypertension was associated with: higher maternal age, Black or mixed ethnicity, higher median weight in early pregnancy, higher systolic and diastolic blood pressure in early pregnancy, and development of pre‐eclampsia (PE) or gestational hypertension (GH) in the index pregnancy. The area under the receiver‐operating‐characteristics curve for the prediction of postpartum hypertension was 0.72 (95% CI, 0.68–0.77) without inclusion of development of PE or GH, and 0.74 (95% CI, 0.70–0.78) with their inclusion. The corresponding detection rates for postpartum hypertension were 49.5% and 53.7%, respectively, at a 20% false‐positive rate. Notably, women with GDM in the index pregnancy frequently had dysglycemia (351/678 (51.8%)) as well as body mass index ≥ 30 kg/m^2^ (232/678 (34.2%)), waist‐to‐height ratio > 0.5 (502/678 (74.0%)), dyslipidemia (186/678 (27.4%)) and/or renal dysfunction (78/678 (11.5%)). At the 5‐month postpartum review, among women with previous GDM who developed postpartum hypertension compared to those who were normotensive at follow‐up, median BMI was significantly higher (30.1 (IQR, 26.7–36.8) *vs* 27.0 (IQR, 23.5–30.6) kg/m^2^), a waist‐to‐height ratio > 0.5 was significantly more prevalent (87.7% *vs* 69.1%) and dyslipidemia was significantly more common (35.8% *vs* 24.4%).

**Conclusion:**

One in four women with recent GDM developed hypertension at 5 months postpartum. However, risk prediction from factors observed in early pregnancy was only modest, reinforcing the need for universal postpartum BP assessment. The high prevalence of hypertension and other cardio‐renal‐metabolic abnormalities following GDM emphasizes the associated enhanced cardiovascular risk, and the postnatal opportunities for intervention early in the life course of these women. © 2026 The Author(s). *Ultrasound in Obstetrics & Gynecology* published by John Wiley & Sons Ltd on behalf of International Society of Ultrasound in Obstetrics and Gynecology.

## INTRODUCTION

Gestational diabetes mellitus (GDM) is a common pregnancy complication and is linked to a higher risk of adverse maternal and neonatal outcomes. With a global prevalence of 14%^1^, women with prior GDM face a 10‐fold increased risk of developing dysglycemia (i.e. prediabetes or Type‐2 diabetes mellitus (DM)^2^), particularly within the first 5 years postpartum^3^.

The focus of GDM management is glycemic control during pregnancy to improve pregnancy outcomes, and glycemic testing postpartum to reduce the risk of undiagnosed Type‐2 DM. However, emerging evidence suggests that women with GDM are also at an increased risk for small‐ and large‐vessel vascular dysfunction^4^, cardiovascular disease and metabolic syndrome and its components, including hypertension^5^, ^6^, ^7^, in the postpartum period. This was highlighted in a recent meta‐analysis of 15 cohort studies^8^ including a total of 3 959 520 patients (175 378 with prior GDM and 3 784 142 without), in which 106 560 cases of postpartum hypertension were reported over a period of 2–20 years, with hypertension occurring more frequently among women with prior GDM.

Hypertension, if left undetected and untreated, is a major risk factor for cardiovascular disease, renal disease and dementia later in life^9^, ^10^, ^11^. Therefore, identifying women at risk for postpartum hypertension is critical for early intervention and implementation of preventative strategies. Previous studies have suggested that factors such as obesity, gestational hypertension and pre‐eclampsia contribute to the risk of postpartum hypertension^12^. However, predictors of postpartum hypertension in women with prior GDM are unknown.

The objectives of this study were, at 5 months postpartum in women with recent GDM, to: (i) evaluate the incidence and determinants of postpartum hypertension; (ii) develop a prenatal prediction model for postpartum hypertension; and (iii) examine the association of postpartum hypertension with other cardiometabolic abnormalities.

## METHODS

### Participant recruitment

At our center (Fetal Medicine Unit, King's College Hospital, London, UK), all pregnant women undergo routine fetal ultrasound at 35 + 0 to 36 + 6 weeks' gestation. From September 2023 to January 2025, all women with GDM attending this ultrasound visit were invited to attend a 5‐month postnatal review clinic. Our strategy aimed to maximize attendance by: (i) informing women of the content and importance of the visit; (ii) providing a wide range of options concerning date and time; and (iii) contacting women 1‐2 days prior to the scheduled visit, to give them the option of changing the date and time of attendance if needed. Patients with chronic hypertension were excluded from this study. Women gave written informed consent for participation. The study was approved by the National Health Service Research Ethics Committee (reference number: 18/NI/0013; Integrated Research Application System ID: 237936).

### Study design and population

This was a single‐center observational prospective cohort study. Our study population comprised individuals with GDM receiving routine maternity care at the Fetal Medicine Unit of King's College Hospital, London, UK, between September 2023 and January 2025. At the 12‐week prenatal visit, data are obtained from patients regarding age, self‐reported ethnicity (White, Black, South Asian, East Asian or mixed), conception method (spontaneous or assisted via *in‐vitro* fertilization or ovulation drugs), medical history (including Type‐1 or Type‐2 pregestational DM), family history of DM (first‐ or second‐degree) and pre‐eclampsia (first‐degree), chronic hypertension, systemic lupus erythematosus, antiphospholipid syndrome and obstetric history (parous or nulliparous (no previous pregnancies ≥ 24 weeks), and if parous, prior GDM or pre‐eclampsia). Assessment includes measurement of maternal weight (in kg), height (in m), body mass index (BMI) (in kg/m^2^) and blood pressure (BP), using an automated device and standardized protocol of three measurements in each arm, with BP taken as the average of the last two measurements in the arm with higher readings^13^. Additionally, ultrasound examination is performed to determine gestational age using fetal crown–rump length^14^ and for diagnosis of major abnormalities.

In the UK, the National Institute for Health and Care Excellence (NICE) guidance^15^ advises that pregnant women undergo risk assessment for GDM in the first trimester. Women with prior GDM should be offered either an early pregnancy 75‐g 2‐h oral glucose tolerance test (OGTT) or blood glucose self‐monitoring. Women with one or more of the following characteristics were offered biochemical testing, usually by OGTT at 24–28 weeks' gestation: ethnicity associated with a high prevalence of GDM (e.g. South Asian, Black), BMI ≥ 30 kg/m^2^, prior pregnancy complicated by macrosomia or GDM, or a first‐degree relative with DM^15^. Once GDM is diagnosed, women are provided with education and intervention to reduce blood glucose levels.

Data on pregnancy outcome were obtained from the computerized maternity records or from the general medical practitioners of the women. Socioeconomic status was measured according to the Index of Multiple Deprivation (IMD) quintile, assigned according to the home postcode of each patient^16^. Information collected includes details of GDM diagnosis and treatment (gestational age at diagnosis and glycemic control with metformin, insulin and/or diet alone), other pregnancy complications and treatment (e.g. gestational hypertension or pre‐eclampsia) and other pregnancy outcomes (e.g. gestational age at birth, birth weight and birth‐weight percentile, according to the Fetal Medicine Foundation charts^17^). Hypertensive disorders of pregnancy were defined using the American College of Obstetricians and Gynecologists (ACOG) criteria^18^. Hypertension was defined as systolic BP ≥ 130 mmHg or diastolic BP ≥ 80 mmHg, on at least two occasions, 4 h apart. Chronic hypertension was defined as hypertension documented before pregnancy or < 20 weeks' gestation^18^. Gestational hypertension was defined as new‐onset hypertension at ≥ 20 weeks' gestation in a previously normotensive woman^19^. Pre‐eclampsia was defined as chronic or gestational hypertension, with development of at least one of the following: new‐onset proteinuria (≥ 300 mg in a 24‐h urine collection or a spot urine protein/creatinine ratio ≥ 0.3 mg/mg), serum creatinine > 97 μmol/L in the absence of underlying renal disease, serum transaminases more than twice the normal limit (≥ 65 IU/L), platelet count < 100 000/μL, headache or visual symptoms, or pulmonary edema^19^.

### Postnatal visit

At the 5‐month postnatal visit, we recorded information on current medications (including those for hyperglycemia, hypertriglyceridemia, hypercholesterolemia or hypertension), weight (in kg), waist circumference (in cm), height (in m) and BP (in mmHg). BP was taken according to a standardized protocol^13^. BMI was classified as normal (18.5–24.9 kg/m^2^), overweight (25.0–29.9 kg/m^2^), obesity Class I (30.0–34.9 kg/m^2^), obesity Class II (35.0–39.9 kg/m^2^) or obesity Class III (≥ 40.0 kg/m^2^). Excess/abnormal adiposity was defined as a waist‐to‐height ratio > 0.5, to adjust waist circumference for general body size, as it is a factor strongly associated with visceral adiposity and the simplest predictor of hypertension, DM and cardiovascular disease^20^.

Women were asked to attend the visit having fasted (no caloric intake for at least 8 h). We performed a 75‐g 2‐h OGTT and measured hemoglobin A1c (HbA1c) (in mmol/mol), serum triglycerides (in mmol/L), serum high‐density lipoprotein (HDL) cholesterol (in mmol/L), serum creatinine (in μmol/L) and urine albumin/creatinine ratio (uACR) (in mmol/mol). All laboratory assays were performed according to standard operational procedures. Serum low‐density lipoprotein (LDL) cholesterol (in mmol/L) was calculated using the Friedewald equation: LDL cholesterol = total cholesterol – HDL cholesterol – (serum triglycerides/5)^21^.

### Outcome measures

The primary outcome was hypertension^19^ at 5 months postpartum, defined as Stage‐1 hypertension (systolic BP 130–139 mmHg or diastolic BP 80–89 mmHg), Stage‐2 hypertension (systolic BP ≥ 140 mmHg or diastolic BP ≥ 90 mmHg) or receipt of antihypertensive treatment.

Other outcomes were obesity, dysglycemia (prediabetes or Type‐2 DM), dyslipidemia and renal dysfunction. Prediabetes was defined according to the criteria of the American Diabetes Association^22^, as HbA1c of 39–47 mmol/mol, fasting plasma glucose (FPG) of 5.6–6.9 mmol/L or impaired glucose tolerance (2‐h blood glucose during OGTT of 7.8–11.0 mmol/L). Type‐2 DM was defined using the World Health Organization (WHO) criteria as HbA1c ≥ 48 mmol/mol, FPG ≥ 7.0 mmol/L, 2‐h blood glucose during OGTT of ≥ 11.1 mmol/L or random plasma glucose ≥ 11.1 mmol/L in a patient with symptoms of hyperglycemia^23^. Dyslipidemia was defined as high triglycerides (≥ 1.7 mmol/L) or use of triglyceride‐lowering treatment, and/or low serum HDL cholesterol (< 1.2 mmol/L) or use of cholesterol‐lowering treatment^24^. Renal dysfunction was defined conservatively as both clinically significant albuminuria (uACR ≥ 3 mg/mmol) and a reduction in estimated glomerular filtration rate (eGFR) to < 90 mL/min/1.73m^2^, calculated using the 2009 Chronic Kidney Disease Epidemiology Collaboration equation^25^, ^26^.

### Statistical analysis

Descriptive statistics were calculated, with continuous variables presented as median (interquartile range (IQR)) and categorical variables as *n* (%). Outcomes were compared between groups using the median test for continuous variables and the chi‐square test for categorical variables.

Backward multivariable logistic regression was performed to assess which factors contributed to postpartum hypertension after GDM. Prior to the regression analysis, continuous variables, such as weight and BMI, were centered by subtracting the median from each value. In the first step, we included maternal demographic characteristics, medical history variables and pregnancy outcomes. In the second step, backward stepwise logistic regression was performed using biologically relevant variables and variables that were significant at the *P* < 0.1 level in the backward elimination step. The relative effect of each variable was expressed as an odds ratio (OR) with 95% CI. The calibration of the model was evaluated using the Hosmer–Lemeshow test (with *P* < 0.05 indicating a poor fit of the model to the data) and its discrimination was evaluated using receiver‐operating‐characteristics (ROC) curves.

All statistical tests were two‐sided. *P* < 0.05 was considered statistically significant. The statistical software SPSS version 29 (IBM Corp., Armonk, NY, USA) was used for data analysis.

## RESULTS

Of 912 consecutive women with GDM who were invited for postnatal clinic review during the study period, 696 (76.3%) attended the follow‐up. Following the exclusion of 18 women with chronic hypertension, our study cohort consisted of 678 women with GDM in their most recent pregnancy. Postnatal follow‐up occurred at a median of 5.1 (IQR, 4.4–6.7) months after birth.

### Characteristics of study population

The characteristics of the study population at 12 weeks' gestation in the index pregnancy, according to development of postpartum hypertension, are presented in Table 1. Overall, most women were in their mid‐30s (median, 35.2 (IQR, 31.9–38.3) years) and more than half (51.8%) self‐identified as belonging to a minority ethnic group. All IMD quintiles were represented. Few women (< 1%) were smokers. Just under half of the population (44.8%) was nulliparous, 107 (15.8%) were parous with previous GDM and 21 (3.1%) were parous with previous pre‐eclampsia. At the 12‐week prenatal examination, the average BMI of the women was in the overweight range, with 232 (34.2%) having a BMI ≥ 30 kg/m^2^, 62 (9.1%) women had systolic BP ≥ 130 mmHg and 96 (14.2%) women had diastolic BP ≥ 80 mmHg. Women who went on to develop postpartum hypertension were more likely to be obese (50.3% *vs* 28.5%) and had a higher median systolic (121.8 *vs* 115.5 mmHg) and diastolic (74.5 *vs* 70.3 mmHg) BP in the early stages of pregnancy. Those who attended the 5‐month follow‐up visit had similar characteristics to those who did not, with the exception that they were of lower socioeconomic status (IMD decile, 3.0 *vs* 5.0), more likely to have a family history of DM (53.4% *vs* 42.6%) and less likely to be parous with previous GDM (28.6% *vs* 38.8%), compared to non‐attenders (Table S1).

**Table 1:** Baseline characteristics at 12 weeks' gestation of women with gestational diabetes melilites (GDM) in index pregnancy, according to hypertension status at 5 months postpartum

Variable	All (*n* = 678)	Postpartum normotension (*n* = 499)	Postpartum hypertension* (*n* = 179)	*P*
Age (years)	35.2 (31.9–38.3)	35.0 (31.7–38.1)	35.4 (32.0–38.8)	0.223
Ethnicity				< 0.001
White	327 (48.2)	245 (49.1)	82 (45.8)	
Black	142 (20.9)	85 (17.0)	57 (31.8)	
South Asian	122 (18.0)	102 (20.4)	20 (11.2)	
East Asian	53 (7.8)	45 (9.0)	8 (4.5)	
Mixed	34 (5.0)	22 (4.4)	12 (6.7)	
Index of multiple deprivation decile				0.730
Quintile 1 (most deprived)	117 (17.3)	81 (16.2)	36 (20.1)	
Quintile 2	120 (17.7)	88 (17.6)	32 (17.9)	
Quintile 3	141 (20.8)	103 (20.6)	38 (21.2)	
Quintile 4	141 (20.8)	105 (21.0)	36 (20.1)	
Quintile 5 (least deprived)	159 (23.5)	122 (24.4)	37 (20.7)	
Smoker	2 (0.3)	1 (0.2)	1 (0.6)	0.448
SLE/APS	6 (0.9)	5 (1.0)	1 (0.6)	0.587
Family history				
PE	26 (3.8)	16 (3.2)	10 (5.6)	0.155
DM	362 (53.4)	273 (54.7)	89 (49.7)	0.251
Parity				0.357
Nulliparous	304 (44.8)	229 (45.9)	75 (41.9)	
Parous	374 (55.2)	270 (54.1)	104 (58.1)	
Previous GDM	107 (28.6)	76 (28.1)	31 (29.8)	0.621
Previous PE	21 (5.6)	14 (5.2)	7 (6.7)	0.549
Method of conception				0.374
Spontaneous	600 (88.5)	439 (88.0)	161 (89.9)	
Ovulation induction	5 (0.7)	5 (1.0)	0 (0)	
*In‐vitro* fertilization	73 (10.8)	55 (11.0)	18 (10.1)	
Weight (kg)	72.6 (62.0–85.1)	69.3 (60.0–81.9)	79.3 (68.0–94.9)	< 0.001
Height (cm)	162.0 (158.0–167.0)	162.0 (157.0–166.0)	162.0 (158.0–167.5)	0.351
BMI (kg/m^2^)	27.4 (23.5–32.6)	26.4 (22.9–31.0)	29.6 (26.0–36.1)	< 0.001
≥ 30 kg/m^2^	232 (34.2)	142 (28.5)	90 (50.3)	< 0.001
Systolic BP (mmHg)	117.0 (110.5–124.3)	115.5 (109.0–121.8)	121.8 (115.3–129.0)	< 0.001
≥ 130 mmHg	62 (9.1)	22 (4.4)	40 (22.3)	< 0.001
Diastolic BP (mmHg)	71.3 (66.5–76.3)	70.3 (66.0–75.0)	74.5 (68.8–80.5)	< 0.001
≥ 80 mmHg	96 (14.2)	46 (9.2)	50 (27.9)	< 0.001

Data are given as median (interquartile range) or *n* (%).

* Defined as systolic blood pressure (BP) ≥ 130 mmHg, diastolic BP ≥ 80 mmHg or receiving antihypertensive treatment. APS, antiphospholipid syndrome; BMI, body mass index; DM, diabetes mellitus; PE, pre‐eclampsia; SLE, systemic lupus erythematosus.

Table 2 presents outcome data of the index pregnancy, and the characteristics of the women at the 5‐month postpartum study visit. GDM was diagnosed < 24 weeks in 128 (18.9%) women, and over half (60.2%) of the study population required metformin with or without insulin. By 36 weeks' gestation, the average BMI of the cohort had increased to Class‐I obesity. Gestational hypertension developed in 34 (5.0%) women and pre‐eclampsia developed in 26 (3.8%) women.

**Table 2:** Outcome data and postpartum findings in study population of women with gestational diabetes mellitus (GDM) in index pregnancy

Variable	All (*n* = 678)	Postpartum normotension (*n* = 499)	Postpartum hypertension* (*n* = 179)	*P*
Pregnancy outcomes				
Diagnosis of GDM < 24 weeks	128 (18.9)	88 (17.6)	40 (22.3)	0.171
Treatment for GDM				0.691
Diet	270 (39.8)	200 (40.1)	70 (39.1)	
Metformin	264 (38.9)	197 (39.5)	67 (37.4)	
Insulin ± metformin	144 (21.2)	102 (20.4)	42 (23.5)	
Weight at 36 weeks (kg)	80.4 (70.1–93.2)	78.3 (68.0–90.3)	87.5 (78.2–101.0)	< 0.001
BMI at 36 weeks (kg/m^2^)	30.7 (27.0–35.0)	30.1 (26.4–33.8)	32.6 (29.5–38.6)	< 0.001
Weight gain from 12 to 36 weeks (kg)	7.7 (4.5–11.0)	7.9 (4.7–11.4)	7.1 (4.0–10.6)	0.364
BMI gain from 12 to 36 weeks (kg/m^2^)	3.0 (1.7–4.3)	3.1 (1.9–4.4)	2.8 (1.5–4.0)	0.223
Gestational hypertension	34 (5.0)	19 (3.8)	15 (8.4)	0.016
Pre‐eclampsia	26 (3.8)	9 (1.8)	17 (9.5)	< 0.001
Gestational age at delivery (weeks)	39.0 (38.3–39.6)	39.0 (38.3–39.6)	39.0 (38.0–39.6)	0.871
Birth‐weight percentile	50.6 (22.9–75.0)	49.8 (21.9–73.9)	51.9 (27.2–79.8)	0.570
Postpartum findings				
Time of postpartum visit (months)	5.1 (4.4–6.7)	5.2 (4.5–6.7)	5.0 (4.4–6.7)	0.492
Breastfeeding	420 (61.9)	315 (63.1)	105 (58.7)	0.291
Weight (kg)	73.3 (62.8–85.1)	70.8 (60.8–82.0)	79.8 (69.6–95.2)	< 0.001
BMI (kg/m^2^)	28.0 (24.0–32.3)	27.0 (23.5–30.6)	30.1 (26.7–36.8)	< 0.001
Waist‐to‐height ratio	0.56 (0.50–0.62)	0.55 (0.49–0.60)	0.58 (0.54–0.66)	< 0.001
> 0.5	502 (74.0)	345 (69.1)	157 (87.7)	< 0.001
Systolic BP (mmHg)	115.5 (108.8–123.5)	112.3 (106.8–118.3)	127.0 (121.3–132.8)	< 0.001
≥ 130 mmHg	69 (10.2)	0 (0)	69 (38.5)	< 0.001
Diastolic BP (mmHg)	73.3 (68.3–78.5)	71.0 (66.5–74.8)	82.3 (80.0–86.0)	< 0.001
≥ 80 mmHg	139 (20.5)	0 (0)	139 (77.7)	< 0.001
Antihypertensive medication	3 (0.4)	0 (0)	3 (1.7)	0.004
Dysglycemia	351 (51.8)	249 (49.9)	102 (57.0)	0.104
Prediabetes	322 (47.5)	231 (46.3)	91 (50.8)	0.170
Type‐2 DM	29 (4.3)	18 (3.6)	11 (6.1)	0.272
Dyslipidemia	186 (27.4)	122 (24.4)	64 (35.8)	0.004
HDL cholesterol (mmol/L)	1.5 (1.3–1.8)	1.5 (1.3–1.8)	1.4 (1.2–1.7)	0.301
Serum triglycerides (mmol/L)	0.9 (0.7–1.3)	0.9 (0.6–1.2)	1.0 (0.8–1.4)	0.011
LDL cholesterol (mmol/L)	3.2 (2.7–3.8)	3.1 (2.6–3.7)	3.4 (3.0–3.8)	< 0.001
Renal dysfunction†	78 (11.5)	54 (10.8)	24 (13.4)	0.352
uACR ≥ 3 mg/mmol	77 (11.4)	54 (10.8)	23 (12.8)	0.463
eGFR < 90 mL/min/1.73m^2^	79 (11.7)	51 (10.2)	28 (15.6)	0.052

Data are given as *n* (%) or median (interquartile range).

* Defined as systolic blood pressure (BP) ≥ 130 mmHg, diastolic BP ≥ 80 mmHg or receiving antihypertensive treatment.

† Defined as both urinary albumin‐to‐creatinine ratio (uACR) ≥ 3 mg/mmol and estimated glomerular filtration rate (eGFR) < 90 mL/min/1.73m^2^. BMI, body mass index; DM, diabetes mellitus; HDL, high‐density lipoprotein; LDL, low‐density lipoprotein; ±, with or without.

The postnatal visit was conducted at a median of 5.1 months postpartum, at which time weight was approximately 1 kg more than that at 12 weeks (Table 2). Most women had a BMI ≥ 30 kg/m^2^ (*n* = 232 (34.2%)) and a waist‐to‐height ratio > 0.5 (*n* = 502 (74.0%)). Postpartum hypertension was diagnosed in 179 (26.4%) participants. In addition, 351 (51.8%) women had dysglycemia, 186 (27.4%) had dyslipidemia and 78 (11.5%) had renal dysfunction.

Compared to women who were normotensive at follow‐up, women who developed postpartum hypertension were more often of Black (31.8% *vs* 17.0%; *P* < 0.001) or mixed (6.7% *vs* 4.4%; *P* < 0.001) ethnicity, had a higher median BMI at 12 weeks' gestation (29.6 *vs* 26.4 kg/m^2^; *P* < 0.001) and systolic (121.8 *vs* 115.5 mmHg; *P* < 0.001) and diastolic (74.5 *vs* 70.3 mmHg; *P* < 0.001) BP at 12 weeks' gestation, had a higher median BMI at 36 weeks' gestation (32.6 *vs* 30.1 kg/m^2^; *P* < 0.001) and more frequently developed gestational hypertension (8.4% *vs* 3.8%; *P* = 0.016) and pre‐eclampsia (9.5% *vs* 1.8%; *P* < 0.001) during the index pregnancy (Table 2). At postnatal review, women with postpartum hypertension had higher median BMI (30.1 *vs* 27.0 kg/m^2^; *P* < 0.001) and more often had a waist‐to‐height ratio > 0.5 (87.7% *vs* 69.1%; *P* < 0.001) and dyslipidemia (35.8% *vs* 24.4%; *P* = 0.004) compared to those who were normotensive. There was no significant difference in the prevalence of dysglycemia or renal dysfunction between those with and without postpartum hypertension.

### Prediction of postpartum hypertension

**Figure 1:**
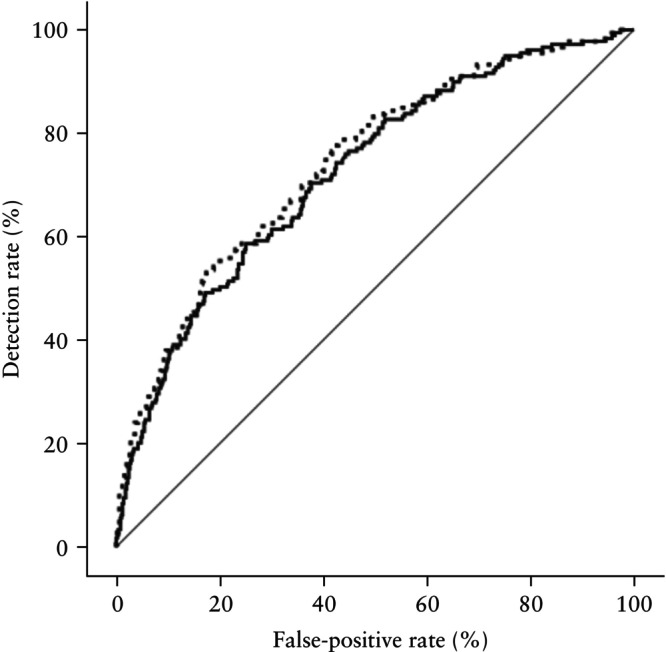
Receiver‐operating‐characteristics curve for detection of postpartum hypertension at 5 months after birth in women with previous gestational diabetes mellitus. Backward multivariable logistic regression model included biologically relevant variables and those that were significant at the *P* < 0.1 level in univariable analysis. 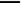, model without development of gestational hypertension (GH) and pre‐eclampsia (PE); 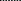, model including development of GH or PE.

In the multivariable analysis (Table 3), postpartum hypertension was associated with: higher maternal age (adjusted OR (aOR), 1.04 (95% CI, 1.00–1.08)); Black (aOR, 1.88 (95% CI, 1.19–3.00)) or mixed (aOR, 2.75 (95% CI, 1.21–6.24)) ethnicity; higher median weight (≥ 73 kg; aOR, 1.57 (95% CI, 1.04–2.38)) and higher systolic and diastolic BP (increment of 1 mmHg; systolic BP: aOR, 1.04 (95% CI, 1.02–1.07); diastolic BP: aOR, 1.04 (95% CI, 1.00–1.07)) at the 12‐week visit for the index pregnancy. Additionally, postpartum hypertension was associated with development of gestational hypertension or pre‐eclampsia (aOR, 3.00 (95% CI, 1.69–5.34)) in the index pregnancy; however, there were few cases of gestational hypertension or pre‐eclampsia, and the 95% CI was wide. Figure 1 shows that ROC curves for the prediction of postpartum hypertension gave an area under the curve (AUC) of 0.723 (95% CI, 0.680–0.766) based only on characteristics available at the 12‐week examination in the index pregnancy, and an AUC of 0.739 (95% CI, 0.697–0.782) when development of gestational hypertension or pre‐eclampsia during the index pregnancy was added to the model. Detection rates for postpartum hypertension were 49.5% and 53.7%, respectively, for the model with and without inclusion of development of gestational hypertension or pre‐eclampsia, at a 20% false‐positive rate. Calibration of the model using the Hosmer–Lemeshow test showed no evidence of poor fit *(P =* 0.857 and *P* = 0.881, respectively).

**Table 3:** Fitted regression model for prediction of postpartum hypertension at the 5‐month visit after birth in women with previous gestational diabetes mellitus (GDM)

Variable	OR (95% CI)	*P*	aOR (95% CI)*	*P*
Baseline characteristics at 12 weeks in index pregnancy				
Age (per 1 year)	1.03 (0.99–1.07)	0.109	1.04 (1.00–1.08)	0.055
Ethnicity		< 0.001	—	—
White	1.00 (ref)	—	—	—
Black	2.04 (1.32–3.05)	0.001	1.88 (1.19–3.00)	0.007
South Asian	0.59 (0.34–1.01)	0.053	—	—
East Asian	0.53 (0.24–1.17)	0.118	—	—
Mixed	1.63 (0.77–3.44)	0.200	2.75 (1.21–6.24)	0.015
Index of multiple deprivation decile		0.731		—
Quintile 1 (most deprived)	1.47 (0.86–2.51)	0.164	—	—
Quintile 2	1.20 (0.69–2.07)	0.515	—	—
Quintile 3	1.22 (0.72–2.05)	0.463	—	—
Quintile 4	1.13 (0.67–1.92)	0.649	—	—
Quintile 5 (least deprived)	1.00 (ref)	—	—	—
Smoker	2.80 (0.17–45.00)	0.468	—	—
SLE/APS	0.56 (0.06–4.78)	0.592	—	—
Parity				
Nulliparous	1.00 (ref)	—	—	—
Parous	1.22 (0.87–1.70)	0.256	—	—
Previous GDM	1.25 (0.76–2.04)	0.382	—	—
No previous GDM	1.15 (0.79–1.67)	0.468	—	—
Previous PE	1.53 (0.59–3.92)	0.380	—	—
No previous PE	1.16 (0.82–1.64)	0.415	—	—
Family history				
PE	1.79 (0.80–4.01)	0.160	—	—
DM	0.82 (0.58–1.15)	0.251	—	—
Method of conception		0.924		
Spontaneous	1.00 (ref)	—	—	—
Ovulation induction	NE	—	—	—
*In‐vitro* fertilization	0.89 (0.51–1.57)	0.691	—	—
Weight (in kg) – 73	2.62 (1.83–3.75)	< 0.001	1.57 (1.04–2.38)	0.034
Height (in cm) – 162	1.21 (0.86–1.71)	0.270	—	—
BMI (in kg/m^2^) – 27	2.53 (1.76–3.64)	< 0.001	—	—
Systolic BP – (Δ1 mmHg)	1.08 (1.05–1.10)	< 0.001	1.04 (1.02–1.07)	0.002
Diastolic BP – ( Δ1 mmHg)	1.09 (1.06–1.12)	< 0.001	1.04 (1.00–1.07)	0.036
Pregnancy outcomes				
Diagnosis of GDM < 24 weeks	1.34 (0.88–2.04)	0.172	—	—
Treatment for GDM		0.691		
Diet	1.00 (ref)	—	—	—
Metformin	0.97 (0.66–1.43)	0.885	—	—
Insulin ± metformin	1.18 (0.75–1.85)	0.480	—	—
Weight at 36 weeks (in kg) – 80	2.48 (1.73–3.56)	< 0.001	—	—
BMI at 36 weeks (in kg/m^2^) – 31	2.60 (1.82–3.71)	< 0.001	—	—
BMI gain from 12 to 36 weeks (in kg/m^2^) – 3	0.79 (0.56–1.11)	0.177	—	—
Gestational hypertension or PE	2.31 (1.15–4.65)	0.019	3.00 (1.69–5.34)	< 0.001

* Backward multivariable logistic regression included biologically relevant variables and those that were significant at the *P* < 0.1 level in univariable analysis. aOR, adjusted odds ratio; APS, antiphospholipid syndrome; BMI, body mass index; BP, blood pressure; DM, diabetes mellitus; OR, odds ratio; NE, not estimable; PE, pre‐eclampsia; ref, reference; SLE, systemic lupus erythematosus; ±, with or without.

## DISCUSSION

### Statement of principal findings

This was a prospective observational study of women with recent GDM, conducted in an ethnically heterogeneous, inner‐city population in South London, UK. We showed that more than 1/4 of women developed postpartum hypertension, observed at postnatal review at a median of 5 months postpartum, using standardized BP measurement. Multivariable analysis showed that significant determinants of postnatal hypertension were identifiable at 12 weeks' gestation (Black or mixed ethnicity, higher median weight and higher systolic and diastolic BP). Their predictive capacity was not meaningfully enhanced by including GDM severity (GDM diagnosis < 24 weeks and/or pharmacological treatment required for GDM control) or the presence of gestational hypertension or pre‐eclampsia in the model, which resulted in a modest AUC of 0.739 with a detection rate for postnatal hypertension of only 53.7% at a false‐positive rate of 20%.

In addition to hypertension, after a pregnancy complicated by GDM, women had an adverse metabolic phenotype at 5 months postpartum. Most women had dysglycemia, as anticipated, but also had an elevated waist‐to‐height ratio. Additionally, over 1/4 of women had dyslipidemia and just over 10% had renal dysfunction. Elevated waist‐to‐height ratio and dyslipidemia were significantly more frequent in women with postnatal hypertension, highlighting the cardiovascular risk of these women.

Our findings emphasize the importance of considering pre‐existing maternal characteristics and early pregnancy maternal phenotype in cardio‐renal‐metabolic risk prediction following GDM, as well as the need for postpartum monitoring of women with prior GDM, not just for dysglycaemia, but for hypertension and other cardiovascular risk factors more broadly.

### Comparison with the literature

Previous studies of the association between GDM and hypertension report conflicting findings; positive associations have been reported in several studies from North America, Europe and Asia^27^, ^28^, ^29^, ^30^, whilst others have reported no association, including the multinational and prospective Hyperglycemia and Adverse Pregnancy Outcome Follow‐Up Study (HAPO FUS)^31^, ^32^, ^33^. In a recent meta‐analysis of 15 cohort studies including 3 959 520 participants (of which 175 378 had previous GDM and 106 560 developed subsequent postpartum hypertension), previous GDM was associated with a 78% increased risk of hypertension at 2–20 years postpartum^8^, albeit with substantial heterogeneity. These findings are consistent with a large, recent population‐based study of 84 746 participants, in which the risk for new chronic hypertension within 24 months postpartum was almost 4‐times higher in women with prior GDM *vs* those without^34^. Consistent with this observation, we showed that hypertension presents early in women with previous GDM, developing in approximately 1/4 of our cohort by 5 months postpartum.

The mechanism for the association between GDM and postnatal hypertension remains unclear. It is possible that our results reflect a causal association (such that lasting metabolic and vascular damage inflicted during a pregnancy complicated by GDM increases the risk that hypertension will develop), or that development of postnatal hypertension among individuals with previous GDM may be mediated through development of pregnancy complications associated with GDM^2^ (e.g. gestational hypertension^35^, pre‐eclampsia^27^ or subsequent Type‐2 DM). However, our findings are most consistent with the link between GDM and postnatal hypertension reflecting shared underlying risk factors for both conditions and for cardio‐renal‐metabolic disease^36^, ^37^. In our univariable and multivariable analyses, development of gestational hypertension or pre‐eclampsia in the index pregnancy did not significantly improve detection of postpartum hypertension, over and above the prediction afforded by maternal characteristics (including Black ethnicity) and early pregnancy BP and weight. The observed link between ethnicity and postpartum hypertension may reflect underlying structural or socioeconomic disparities (although our analyses were adjusted for IMD), or unmeasured factors like discrimination. We have previously reported similar findings in the broader maternity population, among whom the risk of chronic hypertension at 2 years postpartum was predicted most strongly by baseline maternal and early pregnancy characteristics^36^. Additionally, in the Nurses' Health Study II, during 16 years of follow‐up, women with previous GDM had a 26% increased risk of developing hypertension compared with those without a history of GDM, independent of having had gestational hypertension or subsequent development of Type‐2 DM^27^.

Previous research has demonstrated that women at risk of developing GDM have an underlying susceptibility to glucose intolerance (i.e. β‐cell dysfunction and chronic insulin resistance). These defects in insulin sensitivity and secretion are also related to the risk of hypertension^38^. While in our study, early diagnosis of GDM (< 24 weeks' gestation) was numerically (but not statistically) more common among women with postpartum hypertension, previous findings from our group^39^ align with the clustering of obesity, insulin resistance, dyslipidemia and hypertension, increasing long‐term cardiovascular risk. Importantly, this does not align with a narrow post‐GDM focus only on dysglycemia and suggests that these women should be screened for cardiovascular risk factors more generally.

It is noteworthy that we could identify just over 50% of women with previous GDM that later developed postpartum hypertension, even when accounting for BP in early pregnancy, and at a high false‐positive rate (20%). This is insufficient evidence to guide whether BP measurement is required at 5 months postpartum, highlighting the need for structured follow‐up for hypertension and evaluation of excess/abnormal adiposity and other metabolic abnormalities. The inclusion of Black ethnicity as a significant risk factor is a reminder of the importance of considering not only the genetic predisposition of health conditions, but also underlying socioeconomic factors and disparities in healthcare access. Taken together, our findings highlight opportunities for preventative strategies at an early stage of the life course, to address the increased long‐term cardiovascular risk associated with previous GDM. Structured follow‐up could provide comprehensive evaluation and management of cardiometabolic risk through measurement of BP, BMI, waist circumference, glycemic control, lipid profile and renal function, counseling regarding the need for ongoing surveillance and provision of lifestyle advice (Figure S1).

### Strengths and limitations

The strengths of our study include the prospective design, inclusion of a relatively large cohort of women with previous GDM from an ethnically and socioeconomically diverse population, and the high uptake rate of study participation. We undertook detailed phenotyping of all pregnant women seen in our unit from the first trimester, which provided us with the opportunity to assess whether maternal characteristics, BP and development of gestational hypertension or pre‐eclampsia could identify women at risk for postpartum hypertension, as observed previously in the maternity population more generally^36^.

There are limitations of our study that should be considered. Our study cohort of women with GDM was identified following GDM risk‐factor screening and biochemical testing of those at increased risk; this differs from universal biochemical screening or testing employed in other countries. The follow‐up period was limited to a median of just over 5 months, and therefore longer‐term outcomes are unknown. Although follow‐up attendance at 5 months was high, and baseline characteristics did not differ substantially between attenders and non‐attenders, the use of screening based on prior knowledge may have introduced selection bias. In addition, as the study population comprised a specific multiethnic cohort of women with GDM identified by risk factor‐based screening and biochemical testing, the findings may not be generalizable to other populations or settings. Our estimates of the effect of pre‐eclampsia or gestational hypertension on postpartum hypertension were imprecise, given the event rates observed in our cohort. Additionally, we based our diagnosis of postpartum hypertension on BP measurement at one visit, although BP was measured rigorously, according to a standardized protocol that involved multiple measurements from both arms. However, we envision that postpartum surveillance in primary care would provide the opportunity for serial BP monitoring in the clinic or at home. Importantly, the study design precludes establishment of a causal relationship between GDM and postpartum hypertension. Lastly, potential confounders of the relationship between GDM and postpartum hypertension, such as diet and physical activity, could not be accounted for.

### Conclusion

Our study highlights that women with a recent pregnancy complicated by GDM are at high risk for postpartum hypertension, a risk driven primarily by baseline maternal and early pregnancy characteristics rather than the development of gestational hypertension or pre‐eclampsia. Additionally, women with previous GDM have clinically important rates of other cardiometabolic abnormalities, underscoring the need for structured postpartum screening for cardiovascular risk factors beyond dysglycemia and for targeted interventions aimed at reducing cardiovascular risk at this early stage of the life course.

## Supporting information


**Figure S1** Conceptual illustration showing postpartum pathways for structured follow‐up in women with previous gestational diabetes mellitus (GDM). CRM, cardio‐renal‐metabolic; FBG, fasting blood glucose; HbA1c; hemoglobin A1c; LDL‐C; low‐density lipoprotein cholesterol; T2DM, Type‐2 diabetes mellitus; TC, total cholesterol.

**Table S1** Baseline demographic characteristics and pregnancy outcomes of women who declined participation in the 5‐month postnatal review.

## Data Availability

Research data are not shared.
