# Supplementary figures and images for: Hypertension at 5 months postpartum in women with gestational diabetes
Source: Ultrasound Obstet Gynecol. 2026 Jul 17;68(2):202–10. doi: 10.1002/uog.70291 (PMC13432981; doi:10.1002/uog.70291)

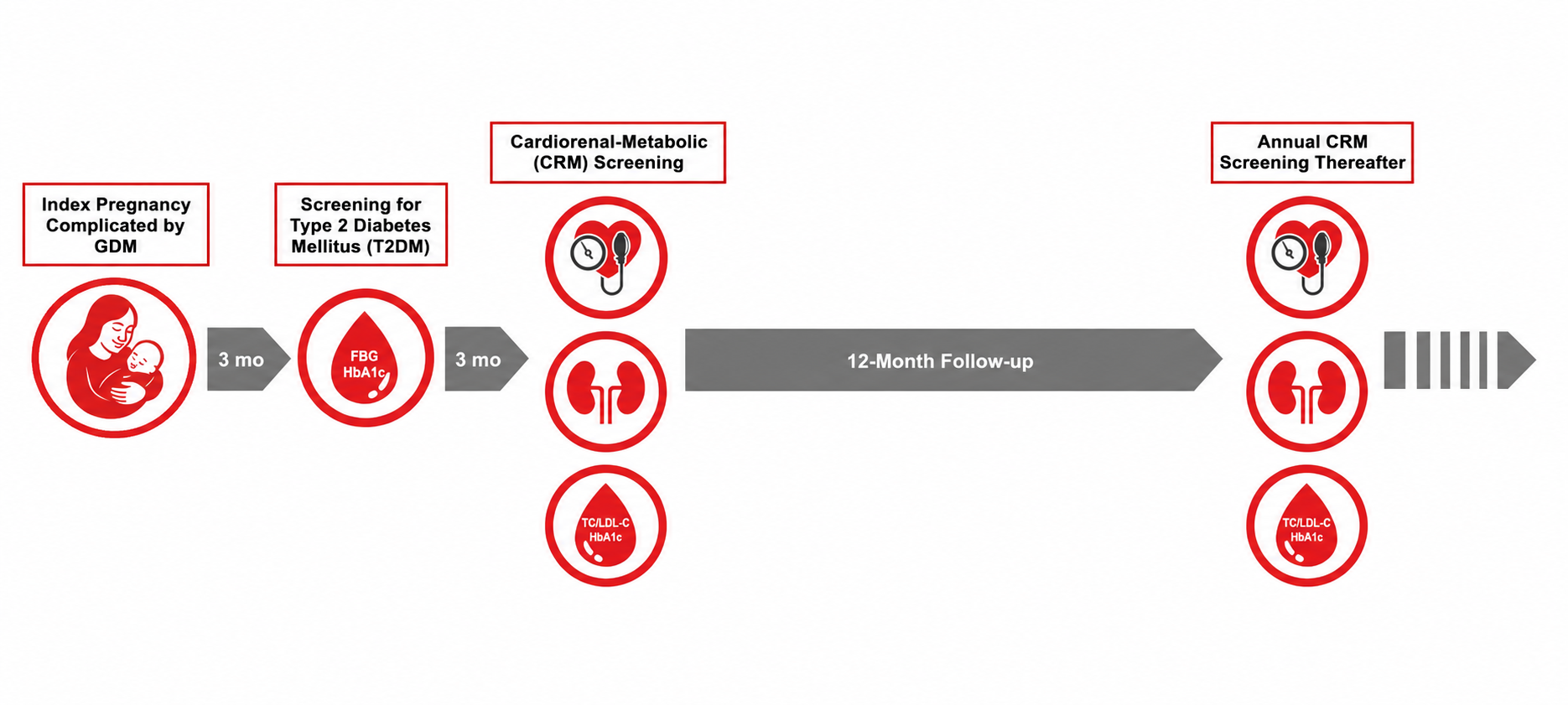

Supplement: Supplementary file 1 — Figure S1 Conceptual illustration showing postpartum pathways for structured follow‐up in women with previous gestational diabetes mellitus (GDM). CRM, cardio‐renal‐metabolic; FBG, fasting blood glucose; HbA1c; hemoglobin A1c; LDL‐C; low‐density lipoprotein cholesterol; T2DM, Type‐2 diabetes mellitus; TC, total cholesterol. [file UOG-68-202-s001.png]
