# Supplementary material for: Hypertension at 5 months postpartum in women with gestational diabetes
Source: Ultrasound Obstet Gynecol. 2026 Jul 17;68(2):202–10. doi: 10.1002/uog.70291 (PMC13432981; doi:10.1002/uog.70291)
Supplement: Supplementary file 2 — Table S1 Baseline demographic characteristics and pregnancy outcomes of women who declined participation in the 5‐month postnatal review. [file UOG-68-202-s002.docx]

**Table S1** Baseline demographic characteristics and pregnancy outcomes for those who declined participation in the 5-month postnatal review.

| **Variable** | **Non-attenders**  (n=216) | **Attenders and included in the analysis ***  (n=678) | **p-value** |
| --- | --- | --- | --- |
| Age in years | 35.0 (31.0-38.0) | 35.2 (31.9-38.3) | 0.139 |
| BMI at 11-13 weeks’ gestation (kg/m^2^) | 28.5 (24.1-32.5) | 27.4 (23.5-32.6) | 0.073 |
| Ethnicity |  |  | 0.067 |
| White | 124 (57.4) | 327 (48.2) |  |
| Black | 38 (17.6) | 142 (20.9) |  |
| South Asian | 38 (17.6) | 122 (18.0) |  |
| East Asian | 9 (4.2) | 53 (7.8) |  |
| More than one | 7 (3.2) | 34 (5.0) |  |
| Index of multiple deprivation decile | 5.0 (3.0-8.0) | 3.0 (2.0-4.0) | <0.001 |
| First or second-degree family history of DM | 92 (42.6) | 362 (53.4) | 0.005 |
| First or second-degree family history of PE | 7 (3.2) | 26 (3.8) | 0.645 |
| Previous pregnancies |  |  | <0.001 |
| Nulliparous | 87 (40.3) | 304 (44.8) |  |
| Parous | 129 (59.7) | 374 (55.2) |  |
| Parous, previous GDM | 50 (38.8) | 107 (28.6) | <0.001 |
| Parous, previous PE | 8 (6.2) | 21 (5.6) | 0.560 |
| BMI at 36 weeks in kg/m^2^ | 30.8 (27.1-34.5) | 30.7 (27.0-35.0) | 0.938 |
| Weight gain in kg | 6.6 (3.8-10.0) | 7.7 (4.5-11.0) | 0.050 |
| Preeclampsia | 10 (4.6) | 26 (3.8) | 0.656 |
| Gestational hypertension | 9 (4.2) | 34 (5.0) | 0.409 |
| Treatment for GDM |  |  | 0.088 |
| Diet | 96 (44.4) | 270 (39.8) |  |
| Metformin | 84 (38.9) | 264 (38.9) |  |
| Insulin (+/- metformin) | 36 (16.7) | 144 (21.2) |  |
| Gestational age at delivery in weeks | 39.0 (38.0-39.5) | 39.0 (38.3-39.6) | 0.908 |
| Birth-weight percentile | 48.1 (22.9-75.7) | 50.6 (22.9-75.0) | 0.815 |

BMI=body mass index, DM=diabetes mellitus, PE=preeclampsia, GDM=gestational diabetes.

* 18 patients diagnosed with chronic hypertension were excluded from the analysis.

Continuous variables are expressed as median (interquartile range) and categorical variables are expressed as n (%).
